# Receptor conversion in metastatic breast cancer: a prognosticator of survival

**DOI:** 10.18632/oncotarget.12114

**Published:** 2016-09-19

**Authors:** Xiangying Meng, Santai Song, Ze-Fei Jiang, Bing Sun, Tao Wang, Shaohua Zhang, Shikai Wu

**Affiliations:** ^1^ Radiotherapy Department, Affiliated Hospital of Academy of Military Medical Sciences, Beijing, China; ^2^ Breast Cancer Department, Affiliated Hospital of Academy of Military Medical Sciences, Beijing, China

**Keywords:** breast cancer, estrogen receptor, progesterone receptor, metastases, survival

## Abstract

**Objective:**

This retrospective study investigated the association between hormone receptor (HR) conversion and survival in breast cancer patients.

**Methods:**

Estrogen receptor (ER) and progesterone receptor (PR) status (positive or negative) of primary tumors and of paired metastatic sites in 627 breast cancer patients were analyzed by McNemar's test for rates of receptor conversion. A survival analysis was performed using the Kaplan-Meier method, and prognostic factors were assessed using Cox's proportional hazards regression model.

**Results:**

Conversion of ER occurred in 165 (26.31%) patients, and conversion of PR in 213 (33.97%; *P* < 0.001, both). For 82 patients whose ER and PR were reassessed 2-4 times during metastatic progression, ER and PR re-conversion occurred in 22 (26.83%) and 29 (35.36%), respectively. The change of ER or PR from positive to negative was associated with worse overall survival and post-recurrent survival (log-rank; *P* < 0.001, both). A subgroup analysis of HR-positive patients (i.e., positive ER, PR, or both) in primary tumor and HR-negative in metastatic sites showed that patients who accepted both salvage endocrine therapy and chemotherapy had better post-recurrent survival than did those who accepted salvage chemotherapy only (log-rank; *P* = 0.003).

**Conclusion:**

ER and PR status may change several times during metastatic tumor progression. A change of HR from positive to negative was associated with worse survival compared with consistent positivity. Repeated evaluations of HR status are necessary in metastatic breast cancer. Salvage hormonal therapy is still worth trying for patients whose HR status changes from positive to negative.

## INTRODUCTION

The 5-year survival rate of women with breast cancer has been improving steadily for the past 5 years, but metastasis to distant sites still limits survival for patients who have undergone radical surgery [1, 2]. Approximately one-third of women with early-stage breast cancer develops distant metastasis and suffers a tumor-related death [3–6].

Endocrine therapy as an adjuvant to prevent and treat metastatic breast cancer is generally well tolerated and efficient. However, the response to endocrine agents such as tamoxifen and aromatase inhibitors, whether partial or complete, is closely dependent on the hormone receptor (HR) status in cancer tissues [7, 8]. Patients whose primary breast tumors are positive for estrogen and progesterone receptors (ER and PR, respectively) are preferred candidates for salvage hormonal therapy, to attenuate progression of the disease.

In routine clinical practice, options for systemic therapy depend on the characteristics of the primary tumor, determined by routine histopathology, immunohistochemistry (IHC), or molecular analysis. However, the choice of treatment may better rest on features of the metastasized lesions rather than that of the primary tumors, in particular with regard to differences in HR status. Differences between the HR status of the primary tumors and metastatic lesions, termed receptor conversion, have been confirmed in 18%-54% of breast cancer patients [9–20]. These results support the necessity for biopsies of metastasized lesions, since the specific HR status of these may alter the choice of therapeutic regimen for these patients [21, 22].

ER and PR levels in primary breast cancers may be important indicators. For example, ER and PR positivity were associated with better treatment outcomes [23], and ER and PR negativity with poorer clinical outcomes [24]. There is some indication from retrospective studies that breast cancer cells that have undergone HR conversion may be more aggressive [25, 26], but a prospective study reported that a therapeutic regimen based on the receptor status of the metastasis did not contribute a survival benefit [10]. Thus the question remains whether identifying the HR status of metastasized breast cancer would aid the choice of therapy strategy.

The present retrospective study of a large cohort of breast cancer patients, compared the ER and PR statuses of primary breast tumors with that of paired metastatic lesions. In addition, we investigated whether HR conversion influenced the survival of these patients, and the effect of HR conversion on salvage hormonal therapy.

## RESULTS

### Patients' clinicopathological characteristics

Six hundred and twenty-seven women with metastatic breast cancer were eligible and involved in our analysis (Table 1). The median ages of the patients at primary tumor diagnosis and metastasis diagnosis were 44 years (range, 22-79 years) and 48 years (range, 25-80 years), respectively.

**Table 1:** Patients' clinicopathological characteristics, *n*/N (%)

		ER		PR	
		Positive	Negative	Positive	Negative
Age, years	<35	76/346 (21.96)	43/281 (15.30)	75/315 (23.81)	44/312 (14.10)
	35-60	252/346 (72.83)	211/281 (75.09)	223/315 (70.79)	240/312 (76.92)
	>60	18/346 (5.20)	27/281 (9.61)	17/315 (5.40)	28/312 (8.97)
Clinical stage	I	70/346 (20.23)	40/281 (14.23)	61/315 (19.37)	49/312 (15.71)
	II	236/346 (68.21)	197/281 (70.11)	212/315 (67.30)	221/312 (70.83)
	III	40/346 (11.56)	44/281 (15.66)	42/315 (13.33)	42/312 (13.46)
Adjuvant therapy	Chemotherapy	317/346 (91.62)	263/281 (93.59)	293/315 (93.02)	287/312 (91.99)
	Hormone therapy	248/346 (71.68)	65/281 (23.13)	218/315 (69.21)	95/312 (30.45)
HER2	Positive^a^	44/346 (12.72)	71/281 (25.27)	47/315 (14.92)	68/312 (21.79)
	Negative^b^	255/346 (73.70)	171/281 (60.85)	226/315 (71.75)	200/312 (64.10)
	NA	47/346 (13.58)	39/281 (13.88)	42/315 (13.33)	44/312 (14.10)
Menstrual status	Menopause	69/346 (19.94)	75/281 (26.69)	56/315 (17.78)	88/312 (28.21)
	Pre-menopause	257/346 (74.28)	186/281 (66.19)	242/315 (76.83)	201/312 (64.42)
	Unknown	20/346 (5.78)	20/281 (7.12)	17/315 (5.4)	23/312 (7.37)

Abbreviations: ER, estrogen receptor; PR, progesterone receptor; HER2, human epidermal growth factor receptor 2; IHC,immunohistochemistry; FISH, fluorescence in situ hybridization; NA, Not Available

a 3+ by IHC or FISH (+)

b 0-2+ by IHC or FISH (−)

In primary breast cancers, the positive rates for ER and PR were 55.18% and 50.24%, respectively. In accordance with the guidelines of the National Comprehensive Cancer Network, positive HR status was defined as positive signs of ER, PR, or both; and negative HR status was defined as negative signs of both ER and PR. In our study, HR positivity was detected in 62.84% (394/627) of all cases.

The biopsied metastatic sites were the following: soft tissues (*n* = 473), liver (*n* = 96), bone (*n* = 21), lung (*n* = 14), ovary (*n* = 9), pleura (*n* = 5), thyroid gland (*n* = 3), brain (*n* = 2), bladder (*n* = 1), stomach (n = 1), kidney (n = 1), and pancreas (*n* = 1). Soft tissues were the most frequent biopsy site of breast cancer metastasis, perhaps because biopsies of soft tissue are safer than of viscera. The next three most common biopsy sites of metastasis were, in descending order, liver, bone, and lung.

### HR conversion

A difference in ER status between the primary breast cancer and metastatic lesions was observed in 165 of 627 patients (26.31%). Specifically, in 106 of 346 patients (30.63%), the ER status had altered from positive to negative, while 59 of 281 patients (21.00%) had an alteration from negative to positive (McNemar's test, *P* < 0.001).

A difference in PR status was observed in 213 of 627 cases (33.97%): in 158 of 315 patients (50.16%) the PR status had changed from positive in primary tumors to negative in the metastatic tissues, while 55 of 312 patients (17.63%) had changed from negative to positive (McNemar's test, *P* < 0.001).

Thus, HR status conversion was detected in 170 of 627 cases (27.11%): 121 of 394 (30.71%) had changed from HR-positive in primary tumors to HR-negative in the metastatic tissues, and 49 of 233 (21.03%) from HR-negative to HR-positive (McNemar's test, *P* < 0.001).

Reassessments were made from multiple biopsies from various organs during tumor progression in 82 patients with advanced breast cancers. The re-conversion rates for ER and PR were 26.83% and 35.36%, respectively (McNemar's test, *P* < 0.05).

Unlike HR status, evidence of human epidermal growth factor receptor 2 (HER2) reflects a more aggressive cancer. The rate of discordance of HER2 between primary tumors and metastases was lower than that of ER and PR: 33 of 503 (6.56%) patients had HER2-negative primary tumors but HER2-positive metastases, and 22 of 503 (4.37%) had HER2-positive primary tumors but HER2-negative metastases (Table 2).

**Table 2:** HER2 status of primary tumor/metastases, by HR status of primary tumor/metastases

HR status ^b^	HER2 status ^a^				
	Prim^+^/Met^+^	Prim^−^/Met^+^	Prim^+^/Met^−^	Prim^−^/Met^−^	Sum
Prim^+^/Met^+^	16/503 (3.18)	14/503 (2.78)	10/503 (1.99)	184/503 (36.58)	224
Prim^−^/Met^+^	4/503 (0.80)	1/503 (0.20)	1/503 (0.20)	31/503 (6.16)	37
Prim^+^/Met^−^	16/503 (3.18)	8/503 (1.59)	4/503 (0.80)	67/503 (13.32)	95
Prim^−^/Met^−^	39/503 (7.75)	10/503 (1.99)	7/503 (1.39)	91/503 (18.09)	147
Sum	75	33	22	373	503

Abbreviations: HR, hormone receptor; HER2, human epidermal growth factor receptor 2; Prim, receptor status of the primary lesion; Met, receptor status of the metastatic lesion.

a HER2 positive: 3+ by IHC or FISH (+); HER2 negative: 0-2+ by IHC or FISH (−)

b HR positive: ER and/or PR positive; HR negative: ER and PR negative.

### Survival analysis

The median follow-up time was 35 months (range, 4-220 months). The median OS (the time from primary breast cancer diagnosis to the date of death or the end of follow-up) and post-recurrent survival (the time from metastatic breast cancer diagnosis to the date of death or the end of follow-up) was calculated on the basis of ER status in the primary tumor and metastatic tissues (Table 3).

**Table 3:** Effects of HR status in primary tumors and metastatic lesions on OS, median months (95% CI)

		*n*	Deaths	OS ^a^	*P*	OS ^b^	*P*
ER	Prim^+^/Met^+^	240	69	135(88-NA)	<0.001 *	68 (44-NA)	<0.001*
	Prim^−^/Met^+^	59	27	85 (55-141)	0.125 **	43 (27-90)	0.142 **
	Prim^+^/Met^−^	106	45	107 (75-156)	<0.001 **	56 (41-83)	0.001 **
	Prim^−^/Met^−^	222	83	73 (42-107)	<0.001 **	39 (28-71)	<0.001 **
PR	Prim^+^/Met^+^	157	48	121 (90-NA)	<0.001*	64 (44-NA)	<0.001*
	Prim^−^/Met^+^	55	26	107 (64-NA)	0.608 **	51 (34-NA)	0.212 **
	Prim^+^/Met^−^	158	59	126 (73-156)	0.133 **	64 (35-89)	0.086 **
	Prim^−^/Met^−^	257	91	79 (45-142)	<0.001 **	41(28-82)	<0.001 **
HR ^c^	Prim^+^/Met^+^	273	86	126 (86-NA)	<0.001 *	64 (43-NA)	<0.001*
	Prim^−^/Met^+^	49	24	105 (62-156)	0.091 **	56 (34-83)	0.095 **
	Prim^+^/Met^−^	121	50	85 (55-122)	<0.001 **	44 (27-90)	0.002 **
	Prim^−^/Met^−^	184	64	73 (42-111)	<0.001 **	39 (28-82)	<0.001 **

Abbreviations: ER, estrogen receptor; PR, progesterone receptor; Prim, receptor status of the primary lesion; Met, receptor status of the metastatic lesion; NA, not available; *n*, number of patients.

a From breast cancer diagnosis to death or censoring.

b From breast cancer metastases to death or censoring.

c HR positive: ER, PR, or both positive; HR negative: ER and PR both negative.

* Compared among 4 groups.

** Compare with Prim^+^/Met^+^ group.

According to the ER status of the primary tumor and metastatic lesions, we divided all 627 breast cancer patients into 4 groups, as follows. Group A: ER-positive in primary tumor and ER-positive in metastatic tissues; Group B: ER-positive in primary tumor and ER-negative in metastatic tissues; Group C: ER-negative in primary tumor and ER-positive in metastatic tissues; and Group D: ER-negative in primary tumor and ER-negative in metastatic tissues.

**Figure 1:**
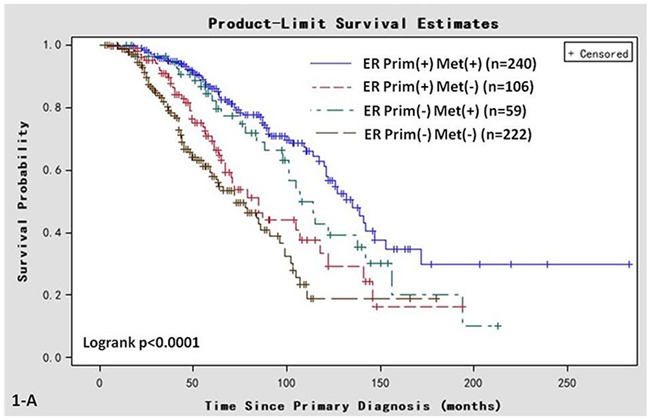

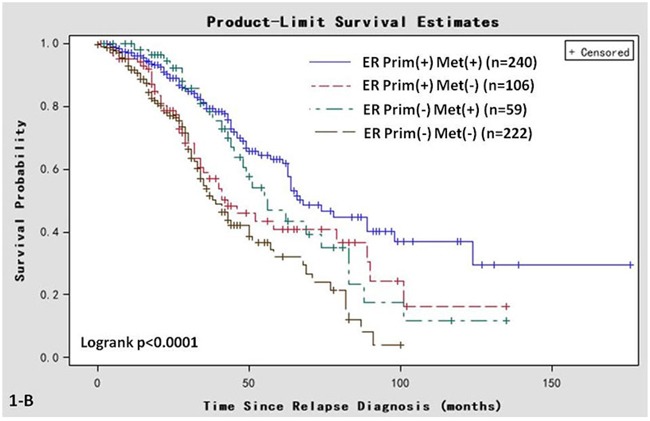
Kaplan-Meier survival curves in women of various ER status subtypes **A.** OS associated with various ER statuses in primary breast cancer (Prim) or metastatic sites (Met). **B.** Post-recurrent survival associated with various ER statuses in primary breast cancer (Prim) and metastatic sites (Met).

There were significant differences in OS (log-rank, *P* < 0.001, Figure 1A) and post-recurrent survival (log-rank, *P* < 0.001, Figure 1B) among the 4 groups. The median OS was 135 (95% CI 88-NA) months in Group A, 85 (95% CI 55-141) months in Group B, 107 (95% CI 75-156) months in Group C, and 73 (95% CI 42-107) months in Group D. The median post-recurrent survival was 68 (95% CI 44-NA) months in Group A, 43 (95% CI 27-90) months in Group B, 56 (95% CI 41-83) months in Group C, and 39 (95% CI 28-71) months in Group D.

According to the PR status of the primary tumor and metastatic lesions, we divided all 627 breast cancer patients into 4 groups, as follows. Group A: PR-positive in primary tumor and PR-positive in metastatic tissues; Group B: PR-positive in primary tumor and PR-negative in metastatic tissues; Group C: PR-negative in primary tumor and PR-positive in metastatic tissues; and Group D: PR-negative in primary tumor and PR-negative in metastatic tissues.

**Figure 2:**
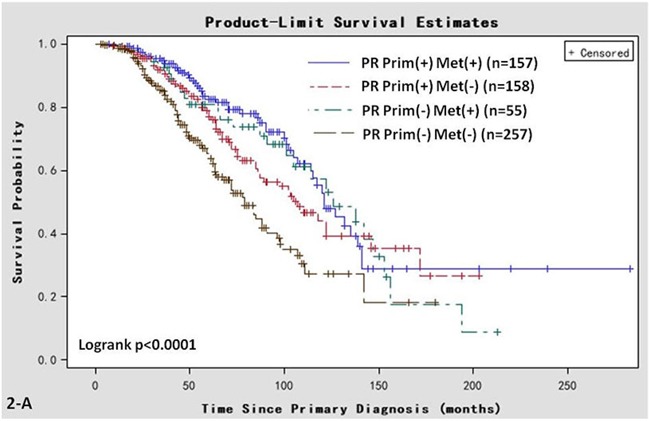

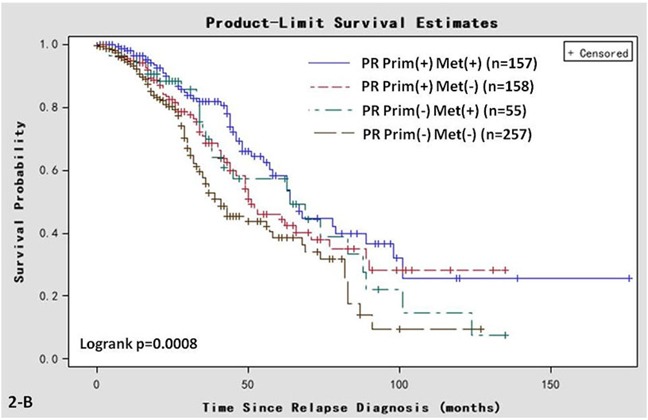
Kaplan-Meier survival curves in women of various PR status subtypes **A.** OS associated with various PR statuses in primary breast cancer (Prim) and/or metastatic sites (Met). **B.** Post-recurrent survival associated with various PR statuses in primary breast cancer (Prim) and metastatic sites (Met).

There were significant differences in OS (log-rank, *P* < 0.001, Figure 2A) and post-recurrent survival (log-rank, *P* < 0.001, Figure 2B) among the 4 groups. The median OS was 121 (95% CI 90-NA) months in Group A, 107 (95% CI 64-NA) months in Group B, 126 (95% CI 73-156) months in Group C, and 79 (95% CI 45-142) months in Group D. The median post-recurrent survival was 64 (95% CI 44-NA) months in Group A, 51 (95% CI 34-NA) months in Group B, 64 (95% CI 35-89) months in Group C, and 41 (95% CI 28-82) months in Group D.

After adjusting for the age of patients with primary breast cancer, age of patients with metastatic breast cancer, PR status in metastatic sites, tumor stage, adjuvant endocrine therapy, adjuvant chemotherapy, and salvage endocrine therapy, the multivariate Cox proportional hazards model was applied. The results of pairwise analysis showed that patients with an ER status that changed from positive in the primary tumor to negative in the metastatic (Prim^+^/Met^−^ group) had a significantly increased hazard ratio for death compared with patients with no change in positive ER status (Prim^+^/Met^+^ group; hazard ratio: 1.74; 95% confidential interval [CI]: 1.19-2.56, *P* = 0.005, Table 4).

**Table 4:** Risks for patients with breast cancer, depending on ER status in primary tumor and metastases

				OS ^a^		Trend test		OS ^b^		Trend test	
		Deaths, *n*		HR ^c^	95% CI	*P*	χ^2^	HR ^c^	95% CI	*P*	χ^2^
ER	Prim^+^/Met^+^	240	69	1.0 ^d^	—	<0.001*	20.81	1.0 ^d^	—	0.004*	13.61
	Prim^−^/Met^+^	59	27	1.28	0.81 to 2.01	0.287 **		1.18	0.75 to 1.86	0.470 **	
	Prim^+^/Met^−^	106	45	1.74	1.19 to 2.56	0.005 **		1.66	1.13 to 2.44	<0.001 **	
	Prim^−^/Met^−^	222	83	2.16	1.53 to 3.05	<0.001 **		1.80	1.28 to 2.53	<0.001 **	
HR	Prim^+^/Met^+^	181	72	1.0 ^d^	—	0.001 *	20.72	1.0 ^d^	—	0.007 *	12.06
	Prim^−^/Met^+^	32	19	1.37	0.86 to 2.18	0.192 **		1.22	0.77 to 1.93	0.404 **	
	Prim^+^/Met^−^	102	52	1.58	1.10 to 2.27	0.013 **		1.48	1.03 to 2.11	0.033 **	
	Prim^−^/Met^−^	125	66	2.21	1.56 to 3.13	<0.001 **		1.78	1.27 to 2.50	<0.001 **	

Abbreviations: ER, estrogen receptor; PR, progesterone receptor; HR, hazard ratio; OS, overall survival; Prim, receptor status of the primary lesion; Met, receptor status of the metastatic lesion; No., number of patients.

Adjusted for age and calendar year of primary breast cancer diagnosis, relapse diagnosis, progesterone receptor status, tumor stage, hormonal treatment, and chemotherapy.

a From breast cancer diagnosis to death or censoring.

b From breast cancer relapse to death or censoring.

c HR positive: ER and/or PR positive; HR negative: ER and PR negative

d Reference

* Compare between 4 groups.

** Compare with Prim^+^/Met^+^ group.

### Salvage hormonal therapy

**Figure 3:**
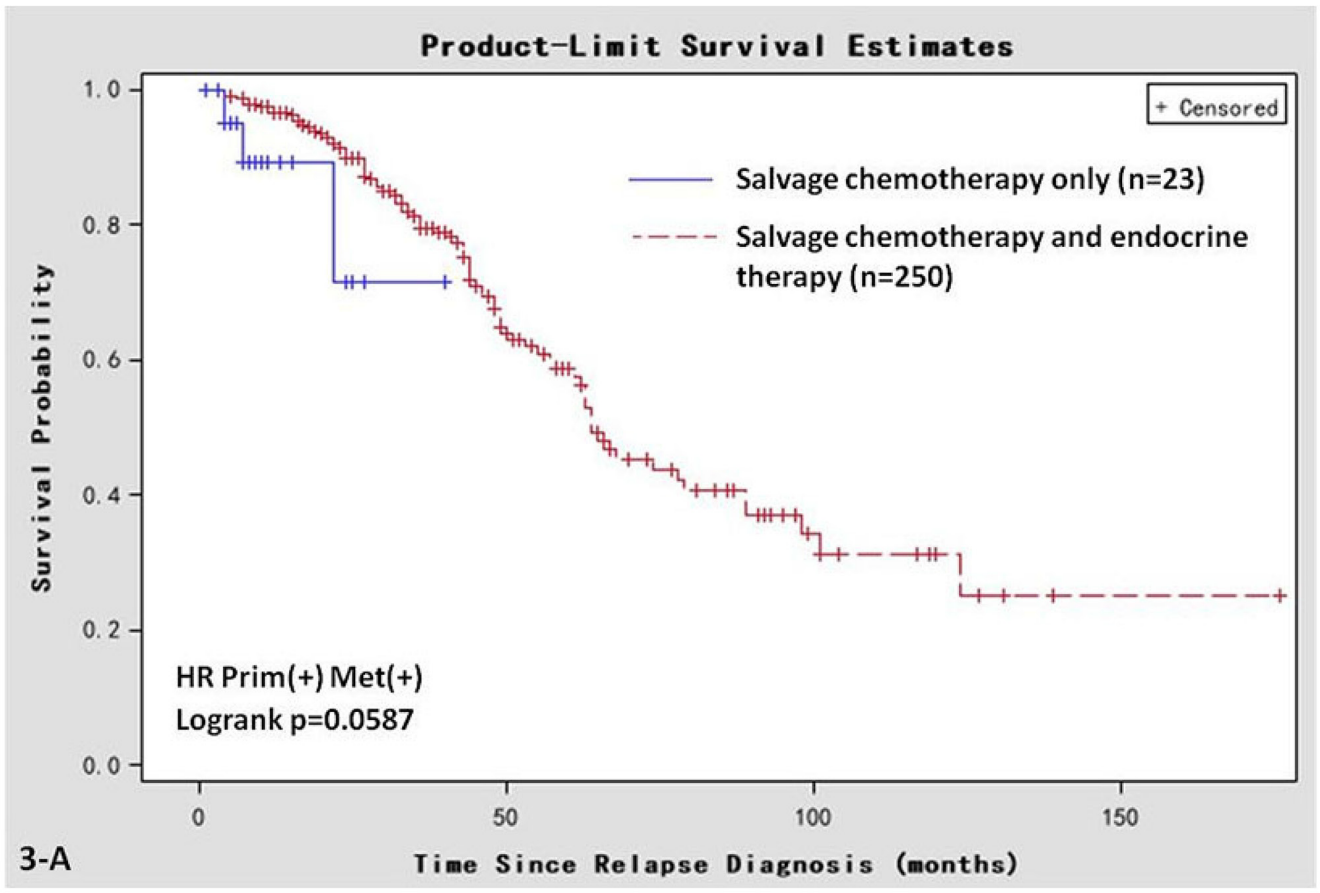

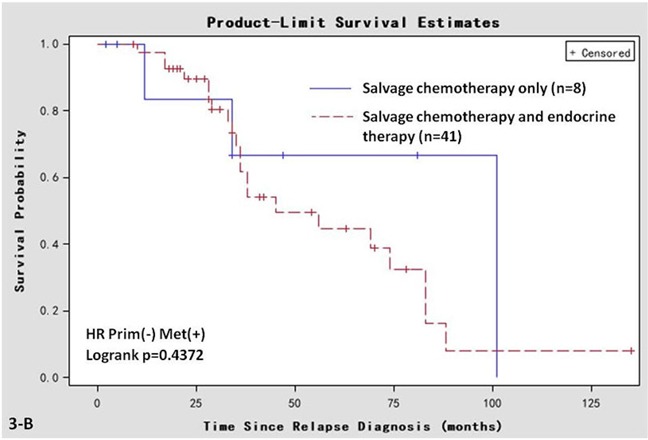

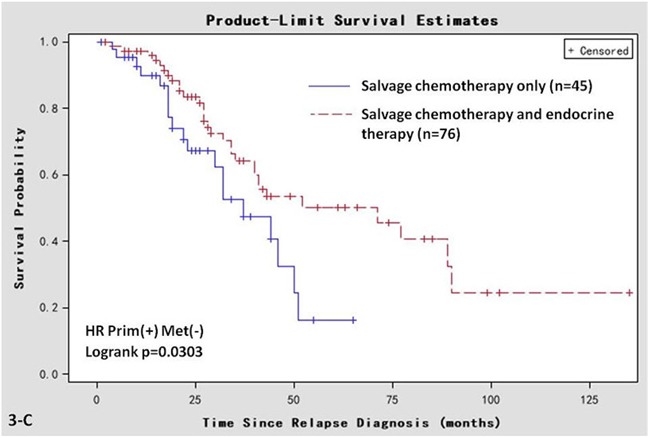

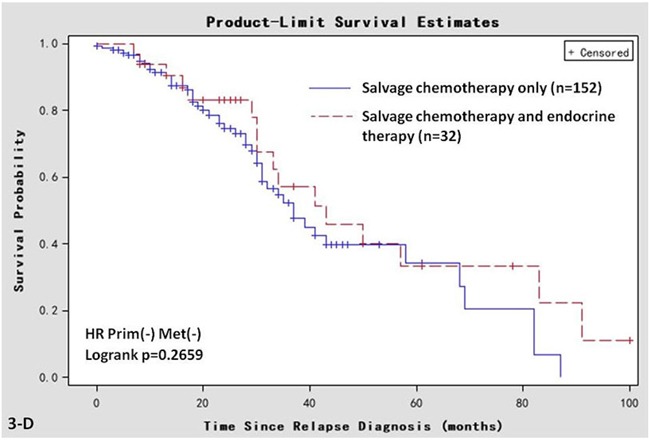
Kaplan-Meier survival curves in women that are HR positive in primary breast cancer and negative in metastatic sites Post-recurrent survival of the cohort receiving both salvage chemotherapy and endocrine therapy, or salvage chemotherapy only. **A.** Prim^+^/Met^+^ group; **B.** Prim^−^/Met^+^ group. **C.** Prim^+^/Met^−^ group; **D.** Prim^−^/Met^−^ group.

A subgroup analysis of this cohort was conducted to investigate the effects of salvage endocrine therapy on breast cancer patients whose HR status changed from positive to negative (121 patients). Of the 121 breast cancer patients in this group, 76 patients received both salvage endocrine therapy and chemotherapy, and 45 patients received salvage chemotherapy only. Univariate Kaplan-Meier analysis was performed to investigate the response to salvage endocrine therapy in terms of post-recurrent survival. We found that the median post-recurrent survival of patients who accepted both salvage endocrine therapy and chemotherapy (71, 95% CI 28-90 months) was better than that of the chemotherapy-only group (37, 95%C I 19-50 months; log rank; *P* = 0.030; Figure 3C).

However, in the Prim^−^/Met^−^ group, the post-recurrent survival of the patients who accepted both salvage chemotherapy and endocrine therapy (*n* = 32) was comparable with the patients who accepted chemotherapy only (*n* = 152; log rank test, *P* = 0.266; Figure 3-D). In the Prim^+^/Met^+^ (Figure 3-A) or Prim^−^/Met^+^ (Figure 3-B) groups, the post-recurrent survival of the patients who accepted both salvage chemotherapy and endocrine therapy was also comparable with the patients who accepted chemotherapy only. However, it is worth noting that most patients with positive HR accepted endocrine therapy. Therefore, the sample size of the chemotherapy-only subgroup was very small (8 patients in the Prim^−^/Met^+^ group and 23 patients in Prim^+^/Met^+^ group). The small sample size restricts the power of these two subgroups to convince.

The multivariate Cox proportional hazards model was applied. After adjusting for the age of patients with primary breast cancer, age of patients with metastatic breast cancer, tumor stage, adjuvant endocrine therapy, adjuvant chemotherapy, clinical phase, and metastatic sites, the results showed that patients with salvage chemotherapy-only had a significantly higher hazard ratio for death compared with patients with both salvage endocrine therapy and chemotherapy (hazard ratio: 1.90; 95% confidential interval [CI]: 1.01-3.58, *P* = 0.045).

With regard to adjuvant endocrine therapy, the age of patients with metastatic breast cancer, tumor stage, adjuvant chemotherapy, clinical phase, and metastatic sites showed no statistically significant association with post-recurrent survival in this multivariate Cox proportional hazards model (Table 5).

**Table 5:** Multivariate Cox proportional hazards model for post-recurrent survival in 121 Prim^+^/Met^−^ patients

	Parameter estimate	Standard error	χ^2^	P*	Hazard ratio	95% CI
Salvage endocrine therapy	0.643	0.322	3.984	0.045	1.902	1.012 to 3.577
Adjuvant endocrine therapy	−0.399	0.297	1.805	0.179	0.671	0.375 to 1.201
Metastatic sites	0.118	0.111	1.129	0.290	1.126	0.905 to 1.400
Adjuvant chemotherapy	−0.732	0.561	1.704	0.192	0.481	0.160 to 1.444
Age at primary diagnosis	0.087	0.078	1.222	0.269	1.091	0.935 to 1.272
Age at relapse diagnosis	−0.101	0.078	1.670	0.196	0.904	0.775 to 1.054
Clinical phase	−0.288	0.203	2.005	0.158	0.750	0.504 to 1.117

**Figure 4:**
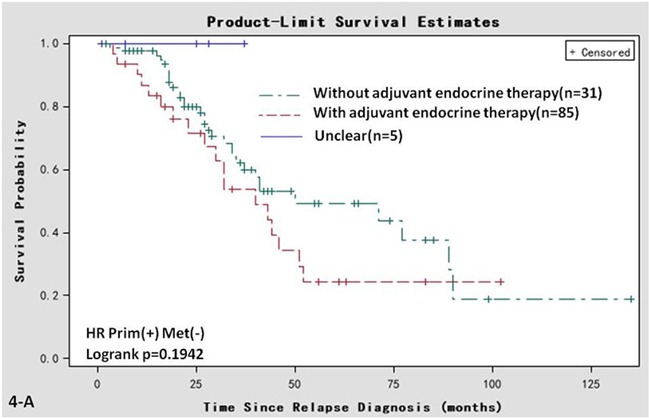

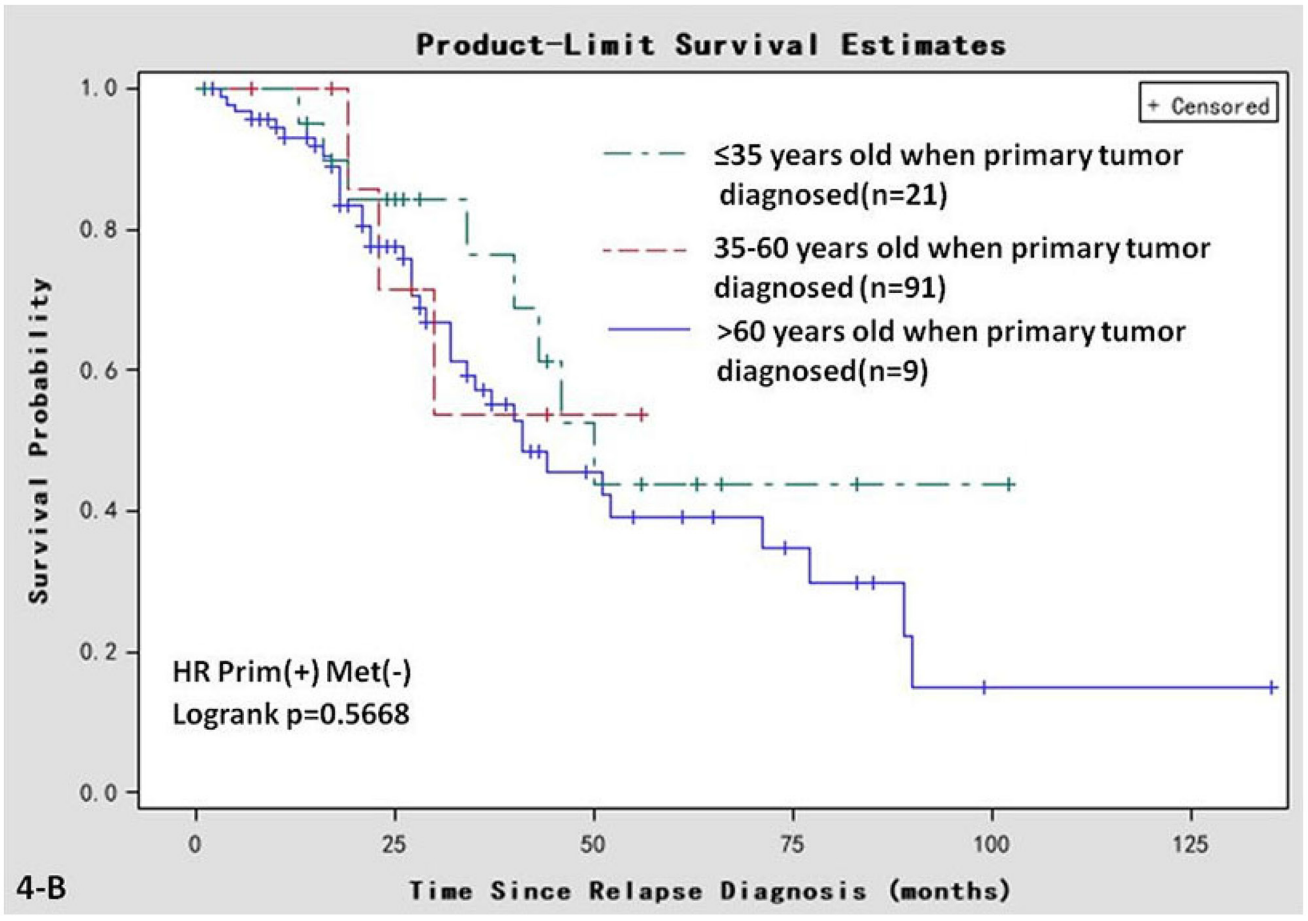

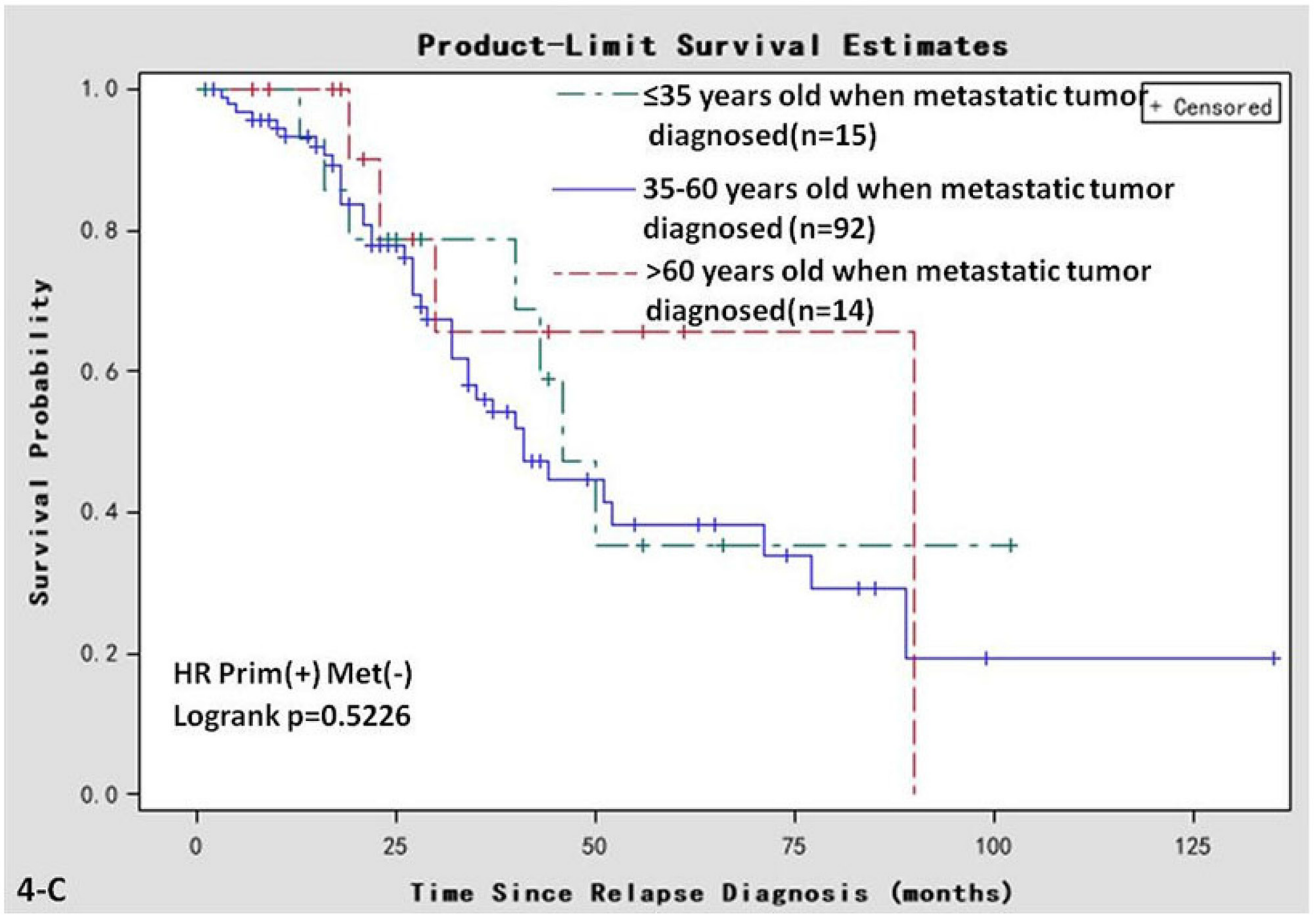

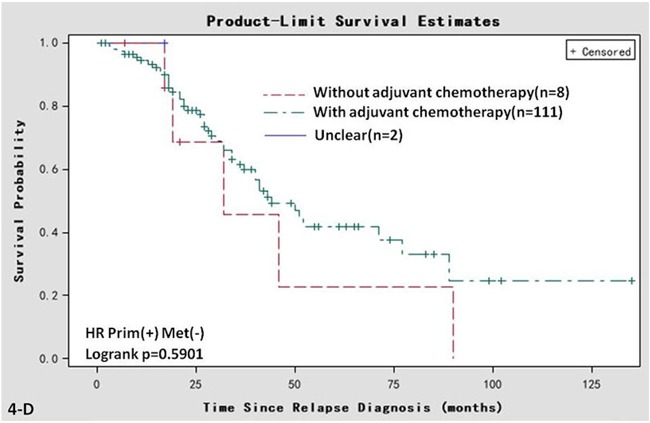

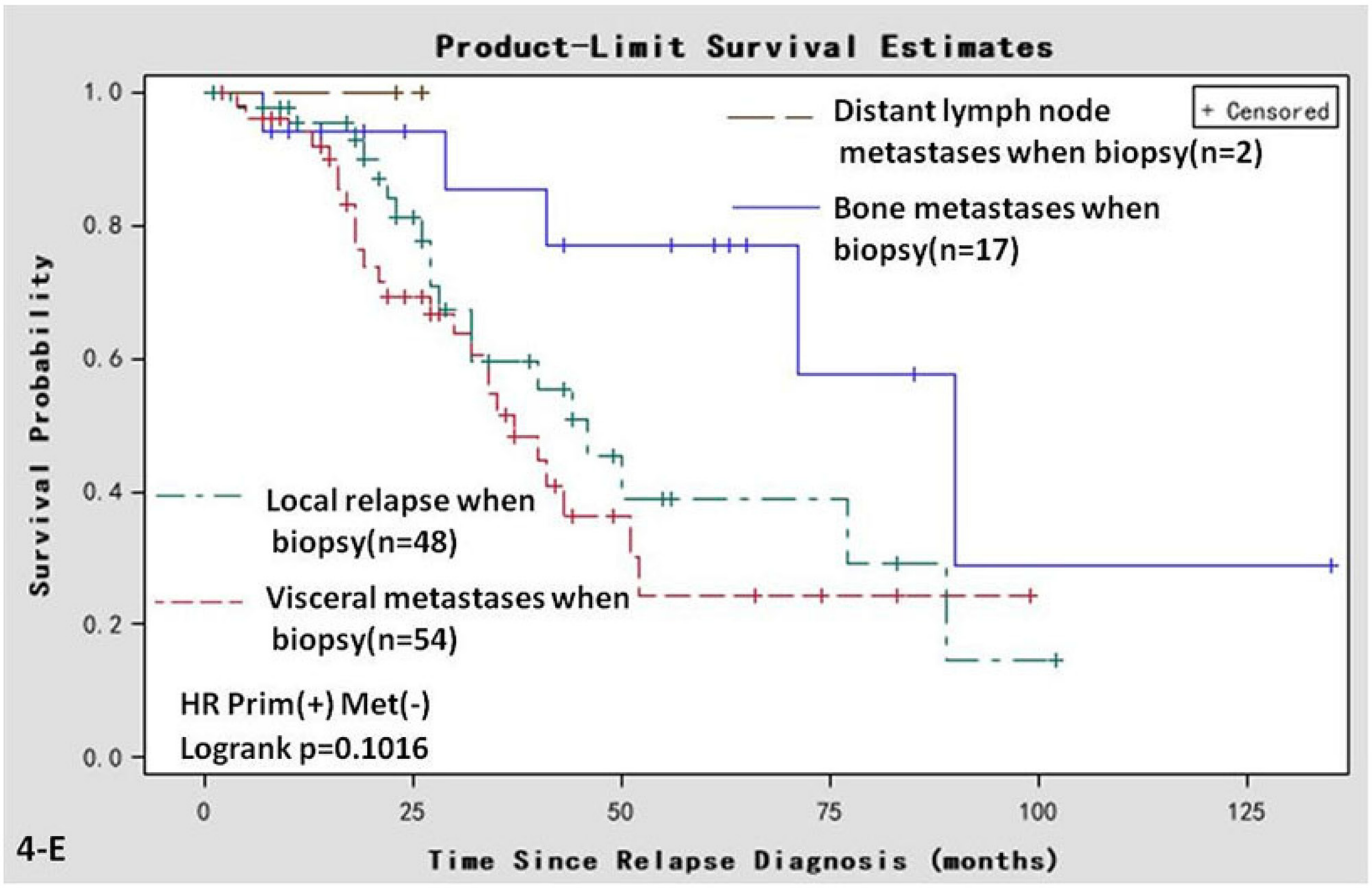

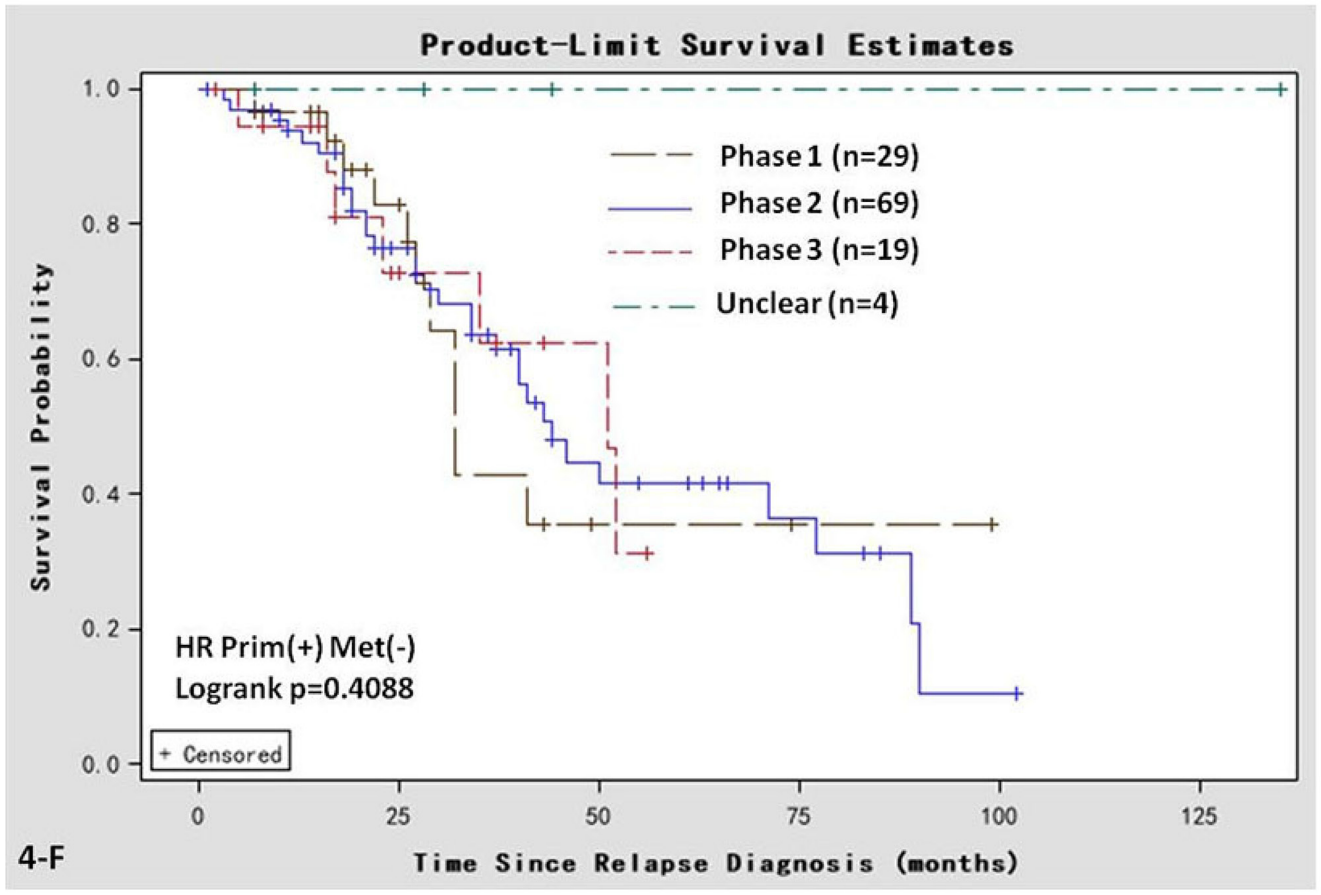
Univariate Kaplan-Meier analyses of post-recurrent survival of 121 patients in the Prim^+^/Met^−^ subgroup **A.** adjuvant endocrine therapy; **B.** age at primary diagnosis. **C.** age at relapse diagnosis; **D.** adjuvant chemotherapy. **E.** metastatic sites; **F.** clinical phase.

Univariate Kaplan-Meier analyses of other parameters were also performed. In the Prim^+^/Met^−^ group, the post-recurrent survival of the patients who had accepted adjuvant endocrine therapy was comparable with that of patients who did not accept adjuvant endocrine therapy (log rank, *P* = 0.194, Figure 4-A). The post-recurrent survival of patients ≤35 years old at diagnosis of the primary tumor was comparable to that of patients aged 35-60 years or >60 years (log rank, *P* = 0.567, Figure 4-B). The post-recurrent survival of patients aged ≤35 years at diagnosis of the metastatic tumor was comparable to that of patients aged 35-60 years old or >60 years (log rank, *P* = 0.523, Figure 4-C). The post-recurrent survival of the patients who had accepted adjuvant chemotherapy was comparable to that of patients who did not receive adjuvant chemotherapy (log rank, *P* = 0.590, Figure 4-D). The differences among the 4 groups of metastatic sites (i.e., distant lymph node metastases; bone metastases; local relapse; and visceral metastases) was not statistically significant (log rank, *P* = 0.102, Figure 4-E). The differences among the clinical phase 1, 2, and 3 groups was not statistically significant (log rank, *P* = 0.409, Figure 4-F).

## DISCUSSION

In this retrospective clinical study, we compared the ER and PR status of primary breast tumors with matched metastases of 627 breast cancer patients, and found evidence that the ER or PR status may be different between primary tumor and metastatic tissues. Furthermore, in some metastatic sites, as the disease progresses, the ER and PR status may change again. We also observed that an ER or PR conversion from positive to negative was negatively associated with OS and post-recurrent survival in these patients. A subgroup analysis of patients with HR-positive status (ER, PR, or both positive) in the primary tumor but HR-negative (ER and PR both negative) in metastatic sites showed that patients who accepted both salvage endocrine therapy and chemotherapy had better post-recurrent survival than did those who accepted chemotherapy only. Systemic therapy prolonged the survival of patients with metastatic breast cancer. HR status in metastatic lesions that differs from that of the primary tumor may have clinical implications in salvage therapy and management.

Several studies have shown that ER and PR were not stable during carcinogenesis and tumor progression [12, 16, 21, 25, 27–38]. In our study, we investigated the conversion of ER and PR in a large cohort of women with breast cancer metastasis. The inclusion and exclusion criteria were chosen to omit bias. We found that the rates of receptor conversion were similar to several other retrospective studies [21, 36, 39]. It is likely that ER and PR conversion results from genetic mutation during tumor progression, intratumoral heterogeneity, and the selective pressure of therapies [29]. A suitable prospective study is needed to determine better the rates of receptor conversion.

According to previous studies, ER and PR conversion in both primary and metastatic lesions in metastatic breast cancer was a prognosticator of OS and post-recurrent survival time [29, 32, 39, 40]. We hypothesize that this correlation can be ascribed to inappropriate target therapy and selection of tumor cells with an unstable phenotype, which may result in more aggressive behaviors [41]. The results of a subsequent univariate analysis showed that a change from positive to negative ER or PR between the primary tumor and metastatic sites was significantly associated with shorter OS compared with no changes. A multi-factorial analysis revealed that only ER status correlated with OS, which was similar to the results of another retrospective study published previously in a peer-reviewed journal [42].

Prospective clinical trials have shown that 14% of patients with breast cancer had their therapeutic regimen modified as a result of changes in the HR/HER2 status in primary tumor tissues or metastatic cancerous tissue despite endocrine treatment before biopsy [10]. In our study, we investigated HR conversion in metastatic breast cancer as a prognostic biomarker during salvage hormonal therapy. Thus, it was of interest that patients whose HR status had altered from positive to negative still achieved a survival benefit from endocrine treatment, compared with those who did not receive endocrine agents. It is likely that HR status in breast cancer tissues will undergo changes several times during tumor progression. In this 627-patient cohort, for 82 patients whose ER and PR were reassessed 2-4 times during metastatic progression, ER and PR re-conversion occurred in 22 (26.83%) and 29 (35.36%), respectively. Thus, our study shows that subsequent and repeated evaluations are necessary for the metastatic breast cancer patient, and salvage hormonal therapy should not be abandoned because of a positive-to-negative HR conversion between the primary tumor and metastatic lesion.

Although we gained some useful insights in our study, its retrospective nature restricts its power to convince. Another shortcoming of our study is a relatively small sample size for the subgroup analysis, especially for the subgroup analysis of the salvage endocrine therapy in the Prim^+^/Met^−^ group. Therefore, a randomized controlled trial is necessary to verify our findings.

In conclusion, ER and PR conversion does occur in breast cancer metastases, and significantly influences survival. Furthermore, ER and PR status may change during tumor progression. Repeated evaluations of HR status are necessary in metastatic breast cancer. Salvage hormonal therapy is still worth trying in cases of positive-to-negative HR conversion.

## MATERIALS AND METHODS

### Patients

We conducted a single-center retrospective clinical study, initially collecting the clinical data of 3674 patients with invasive breast cancer who had been hospitalized sometime between 1 January 2002 and 1 April 2016 at Affiliated Hospital of Academy of Military Medical Sciences, Beijing, China.

The inclusion criteria were: pathologically confirmed breast cancer; metastatic disease biopsy; obtainable status of ER, PR, or both of primary and metastatic tumors; and receiving treatment provided by an oncology team that included an oncosurgeon, a medical oncologist, and a radiologist. Patients' clinicopathological information, including age, gender, date of invasive breast cancer diagnosis, date of metastatic diagnosis, location of distant metastasis, survival data, tumor pathological stage, adjuvant therapy regimen, and salvage therapy regimen was collected and recorded by the oncologists. Excluded from this study were patients without biopsy data on the metastasis with HR status, or patients with clinical stage IV breast cancer, bilateral primary breast cancer, contralateral breast metastasis, and those receiving neoadjuvant chemotherapy before biopsy.

The final analysis included 627 patients, all of whom had been biopsied at both the primary and paired metastatic sites. The academic and ethics committees of our hospital approved this retrospective study. All patients provided written informed consent before the biopsy.

### ER, PR, and HER-2 testing

The primary and metastatic ER and PR statuses of each patient were required for this study and were obtained from the pathology reports. The ER and PR status were both evaluated using immunohistochemistry (IHC) as described previously [43]. Immunohistochemical analysis was carried out on full 4-μm sections. For ER alpha (ERα) and PR, the percentage of positively stained nuclei was estimated. Appropriate negative and positive controls were used throughout. A threshold ≥1% of stained nuclei was considered a positive status. Scoring of IHC slides was performed by 2 independent pathologists, blinded to other data in the paired samples. In primary tumor samples, the adequacy of staining was checked by comparison with normal breast parenchyma of the same patient.

We used 2 methods to assess tissue HER2 status in our study: IHC and fluorescence *in situ* hybridization (FISH). To evaluate HER2 overexpression, IHC staining of specimens was conducted using paraffin-embedded breast cancer tissues and polyclonal rabbit anti-human HER2 oncoprotein. The latter targets the intracellular domain of HER2 protein. Tumors which scored 0 or 1+ were designated HER2-negative; those that scored 3+ were considered HER2-positive. The tumors that were scored via IHC as 2+, were analyzed further by FISH using a PathVysion HER-2 DNA probe kit (Abbott Laboratories, Abbott Park, IL), in accordance with the manufacturer's recommended protocol. A FISH result was defined as positive when the HER2/cep17 ratio was >2.2.

### Statistical analysis

Comparisons of ER and PR statuses between the primary tumor and paired metastatic sites were performed using McNemar's test. Overall survival (OS) was defined as the time from the date of the pathological diagnosis of primary breast cancer to the date of death or at the end of follow-up (1 May 2016). Post-recurrence survival was considered from the date of the pathological diagnosis of metastatic breast cancer to the date of death or at the end of follow-up (1 May 2016). The Kaplan-Meier analysis was applied to determine whether HR conversion could be a prognosticator of survival [44, 45]. The risk of tumor-related death associated with ER and PR status was modeled using a multivariable Cox proportional hazard model. An arbitrary level of 5% was used to indicate statistical significance. Our clinical data was analyzed using SAS version 9.2 statistical software.
